# Genome-Wide Mapping of the Cohesin Complex in the Yeast Saccharomyces cerevisiae

**DOI:** 10.1371/journal.pbio.0020259

**Published:** 2004-07-27

**Authors:** Earl F Glynn, Paul C Megee, Hong-Guo Yu, Cathy Mistrot, Elcin Unal, Douglas E Koshland, Joseph L DeRisi, Jennifer L Gerton

**Affiliations:** **1**Stowers Institute for Medical Research, Kansas CityMissouri, United States of America; **2**Department of Biochemistry and Molecular Genetics, University of ColoradoDenver, Colorado, United States of America; **3**Howard Hughes Medical Institute, Department of EmbryologyCarnegie Institution of Washington, Baltimore, Maryland, United States of America; **4**Department of Biochemistry and Biophysics, University of CaliforniaSan Francisco, CaliforniaUnited States of America

## Abstract

In eukaryotic cells, cohesin holds sister chromatids together until they separate into daughter cells during mitosis. We have used chromatin immunoprecipitation coupled with microarray analysis (ChIP chip) to produce a genome-wide description of cohesin binding to meiotic and mitotic chromosomes of Saccharomyces cerevisiae. A computer program, PeakFinder, enables flexible, automated identification and annotation of cohesin binding peaks in ChIP chip data. Cohesin sites are highly conserved in meiosis and mitosis, suggesting that chromosomes share a common underlying structure during different developmental programs. These sites occur with a semiperiodic spacing of 11 kb that correlates with AT content. The number of sites correlates with chromosome size; however, binding to neighboring sites does not appear to be cooperative. We observed a very strong correlation between cohesin sites and regions between convergent transcription units. The apparent incompatibility between transcription and cohesin binding exists in both meiosis and mitosis. Further experiments reveal that transcript elongation into a cohesin-binding site removes cohesin. A negative correlation between cohesin sites and meiotic recombination sites suggests meiotic exchange is sensitive to the chromosome structure provided by cohesin. The genome-wide view of mitotic and meiotic cohesin binding provides an important framework for the exploration of cohesins and cohesion in other genomes.

## Abstract 2

Chromatin immunoprecipitation reveals that cohesin sites -- those responsible for holding sister chromatids together until they separate into daughter cells -- are conserved in meiosis and mitosis

## Introduction

Sister chromatid cohesion ensures that daughter cells inherit complete copies of their genome. Cohesion in eukaryotic cells is mediated by a multisubunit protein complex called cohesin. Cohesin consists of four proteins: Smc1, Smc3, Scc1/Mcd1, which is called kleisin and is the target of the protease separase, and Scc3. These proteins have recently been proposed to form a ring structure that encircles sister chromatids (Gruber et al. 2003). Alternately, the ring may act as a snap (Milutinovich and Koshland 2003). Cohesion is established during replication and maintained until metaphase in mitosis (Uhlmann and Nasmyth 1998). All members of the cohesin complex are essential in *Saccharomyces cerevisiae,* since mutation results in the precocious dissociation of sister chromatids.

Cohesion serves at least three roles in the cell with respect to accurate genome transmission. Firstly, cohesion proximal to the centromere facilitates biorientation of chromosomes with respect to the spindle (Tanaka et al. 2000). Secondly, it prevents splitting of chromosomes once bipolar attachments are made (Tanaka et al. 2000). Thirdly, cohesin bound along chromosome arms may be essential for proper chromosome condensation in yeast (Guacci et al. 1997). In meiosis, cohesin at most arm sites disappears prior to the first nuclear division. The meiotic cohesin complex contains Rec8 instead of Scc1/Mcd1 (Klein et al. 1999). Cohesion is maintained distal to crossovers between homologs, which links them and facilitates their biorientation on the meiotic I spindle. Cohesin is also maintained at pericentric regions, which is essential for biorientation of chromosomes at the spindle for the second nuclear division (Buonomo et al. 2000).

We are interested in understanding the *cis* determinants of cohesin binding. Cohesin-associated regions have been identified in yeast using chromatin immunoprecipitation. In these studies cohesin association with chromatin was followed at low resolution along the entire length of Chromosome III (3-kb intervals) or high resolution (300-bp intervals) at limited regions on Chromosome III, V, and XII (Blat and Kleckner 1999; Megee et al. 1999; Tanaka et al. 1999; Laloraya et al. 2000). These studies showed associations of cohesin with specific regions of chromosomes; one of the regions of intense association is centromeres. In addition to the enrichment of cohesin in the pericentric region of Chromosome III, Blat and Kleckner (1999) found a spacing of cohesin-associated regions of 13 kb along the arms of Chromosome III. A similar spacing was observed in a limited region of Chromosome XII (Laloraya et al. 2000). These studies also noted a correlation of cohesins with elevated AT content. The average size of the mapped sites was 0.8–1 kb (Laloraya et al. 2000). Based on three sites mapped to high resolution, cohesin was proposed to associate with the boundaries of transcriptionally silent regions (Laloraya et al. 2000).

Despite these insights into *cis* determinants of cohesin binding, many aspects of the cohesin–DNA interaction remain obscure. The high resolution studies sampled a small portion of the genome, and the low-resolution analysis of Chromosome III does not address questions about the position of cohesin relative to smaller-scale genome features, such as individual transcription units. Furthermore, Chromosome III is the sex chromosome of budding yeast, and, similar to other organisms, it has unusual properties including large domains of repressed recombination, silent mating type loci, and different patterns of replication (Reynolds et al. 1989; Wu et al. 1997). Some discrepancies between high- and low-resolution studies have emerged, including, for example, whether cohesin is found at telomeres (Blat and Kleckner 1999; Laloraya et al. 2000).

One approach to better understand *cis* determinants of cohesin binding is to analyze them across the whole genome. To obtain a genome-wide picture of cohesin binding to S. cerevisiae chromosomes at 1–2-kb resolution, we used a combination of chromatin immunoprecipitation (ChIP) and microarray methods, often referred to as ChIP chip technology. To aid identification of peaks of cohesin binding we developed a program, PeakFinder, for extraction of peaks from raw ChIP data. We further used this approach to map all the cohesin-binding sites on an “ectopic chromosome,” a yeast artifical chromosome containing a 334-kb insert from human Chromosome VII. Information from a large number of sites greatly facilitates the assessment of cohesin distribution and of the significance of correlations with many local properties of the genome, such as base composition and coding content. Furthermore, it allows us to evaluate the impact of several factors, such as strain background, transcription, and developmental programs like meiosis, on cohesin binding, and to test the predictions by engineering individual cohesin sites.

## Results

### Determining Sites at Which Cohesin Interacts with the Yeast Genome

We used the genome-wide approach of ChIP chip to identify and evaluate *cis* determinants of cohesin sites (detailed protocol for ChIP available at http://www.uchsc.edu/sm/bbgn/images/ChIP%20protocol.htm; see also Protocol S1). We began the study using Mcd1-18Myc as the protein target in the W303a strain background. Cells were arrested in metaphase by a temperature-sensitive mutation in *CDC16,* a subunit of the anaphase-promoting complex required to degrade Pds1p, a negative regulator of anaphase (Yamamoto et al. 1996; Cohen-Fix and Koshland 1997). We were interested in determining potential correlations between cohesin binding and genome features such as base composition, transcriptional state, and known *cis* determinants of chromosome transmission. Earlier studies have used simple ratio “thresholds” to define binding sites in ChIP chip data (Iyer et al. 2001; Lieb et al. 2001; Wyrick et al. 2001). A single genome-wide threshold would be of limited value in our experiments because (1) peaks representing the intensity of cohesin binding are much higher at pericentric regions than towards the end of chromosomes, therefore, a threshold constant would have the effect of skewing all the binding sites towards the centromere-proximal regions; (2) binding sites in ChIP chip data are frequently defined by several array elements, complicating the identification of *cis* determinants; and (3) much of the analysis has to be done manually. A better approach would be to use the local parameters of cohesin binding to identify the peaks. To aid such a task, we have written a Windows program, PeakFinder, which discerns and filters the peaks from a variable local background and maps the tip of the peak to a single array element. The program is freely available (http://research.stowers-institute.org/jeg/2004/cohesin/peakfinder).

**Figure 1:**
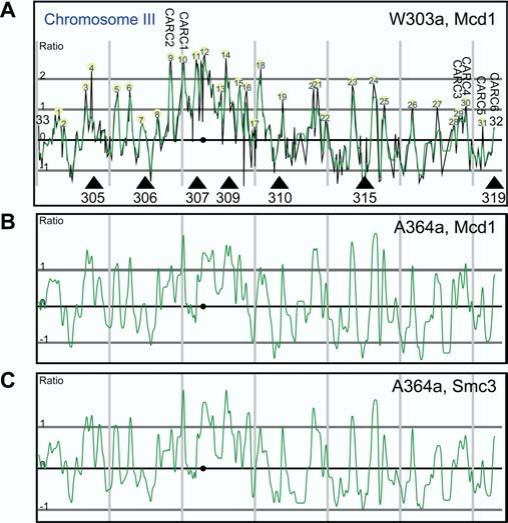
Interactions between Cohesin and CHRIII in S. cerevisiae The centromere is indicated with a black circle; the smoothed data are indicated with a green line. 50-kb intervals are indicated by vertical grey lines. (A) Data generated from a *cdc16*-arrest ChIP for Mcd1-18Myc in W303a. The midpoint of each feature is used to represent the log_2_ of the median red:green ratio (left y-axis) with a black line, high firing replication origins are indicated with black triangles, and previously mapped CARC2, CARC1, CARC3, CARC4, CARC5, and CARC6 (Laloraya et al., 2000) correspond to peaks 9, 10, 29, 30, 31, and 32, respectively. Peaks are located and numbered by PeakFinder (with the exception of telomeres) using the parameters described in the Materials and Methods. (B) Smoothed data from *cdc16*-arrest ChIP for Mcd1/Scc1-6HA in A364a. (C) Smoothed data from *cdc16*-arrest ChIP for Smc3-6Myc in A364a.

**Figure 2:**
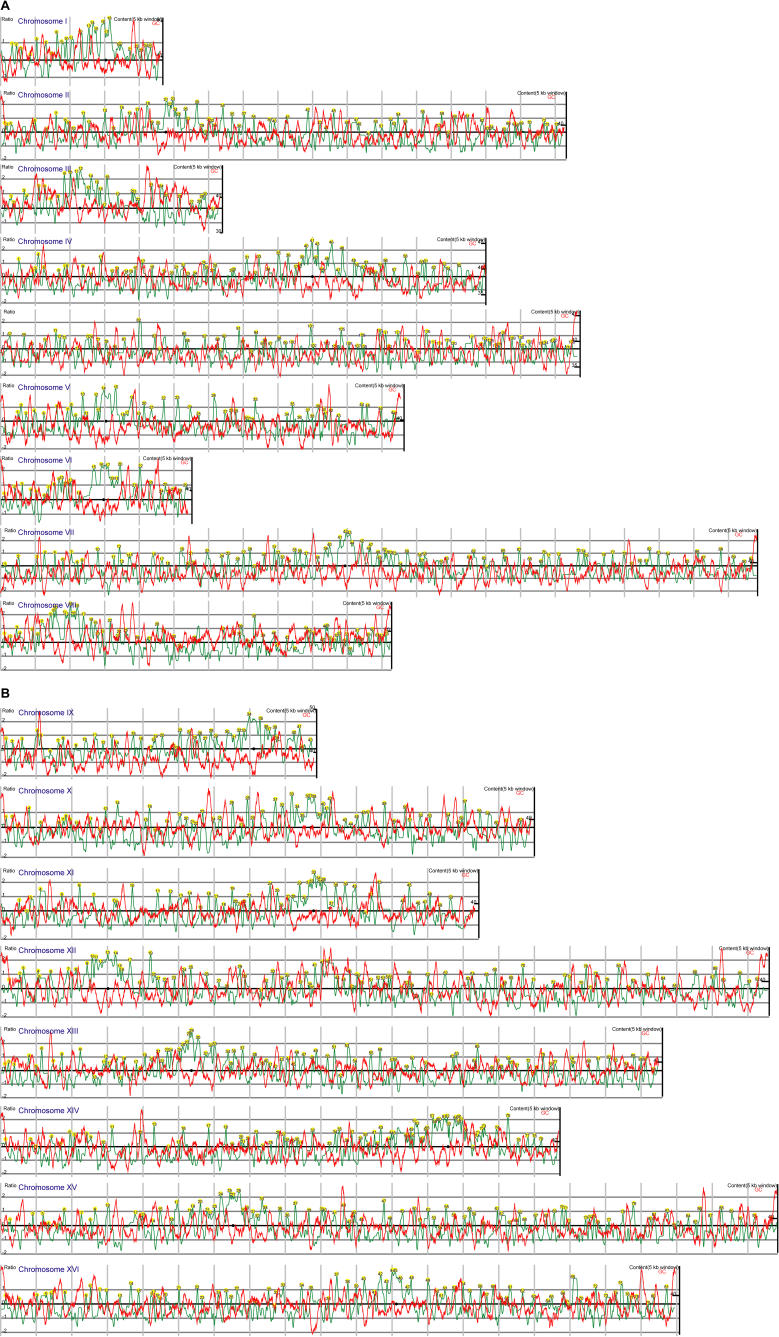
Visual Representation of the Interactions between Mcd1-18Myc and the S. cerevisiae Genome in W303a For each of the 16 chromosomes the centromere is indicated with a black circle, the smoothed data (based on the log_2_ of the ratio) is indicated with a green line (left y-axis), and the percent GC is indicated by a red line (right y-axis). Vertical grey bars mark 50-kb intervals. Peaks are located and numbered by PeakFinder (with the exception of telomeres) using the parameters described in the Materials and Methods. For Chromosome XII, peaks 41 and 42 correspond to the previously described peaks CARL1 and CARL2 (Laloraya et al. 2000).

We validated our methods by comparing the results to previously collected data. Laloraya et al. (2000) discovered nine sites in the arms of Chromosomes III and XII using ChIP followed by semiquantitative PCR. All of those sites could be identified as peaks in the microarray data using PeakFinder (for Chromosome III see Figure 1). Blat and Kleckner (1999) mapped 23 cohesin sites to 3-kb resolution on Chromosome III. Although the number of identified peaks is increased in our data (33 versus 23), the increase can largely be accounted for by higher-resolution mapping. Qualitatively the results are comparable, including that (1) the peak height at *CEN3* is the highest for the chromosome, (2) the height of the peaks declines towards the ends of the chromosome, and (3) peaks correlate with AT content (Figure 2). Therefore, the results of previous studies are reproduced by our methods.

Three additional controls were performed to validate our methods. First, immunoprecipitation from a strain without any epitope tags on the cohesin complex was performed and did not yield any signal (Megee et al. 1999). Second, for each ChIP performed with the anti-Myc antibody, a second ChIP was performed with the same chromatin solution in which the anti-Myc antibody was omitted. The immunoprecipitated DNA was subjected to semiquantitative PCR for centromere sequence (SGD coordinates 114318–114561) on Chromosome III. When the amplification was in the linear range, the difference in signal between the two templates was 11-fold ± SD 3.2, demonstrating that the enrichment for this particular sequence is specific to the interaction between the Myc epitope and the anti-Myc antibody. The ChIP samples were subjected to 20–25 cycles of random PCR amplification (http://microarrays.org/protocols.html; see also Protocol S2) prior to labeling and hybridization to microarrays. This amplification procedure was performed side by side on ChIP samples obtained with and without primary antibody. After 25 cycles of PCR, equal amounts of the samples were loaded on an agarose gel. The sample generated in the absence of primary antibody did not contain any detectable DNA, while the sample obtained with primary antibody generated a robust smear of DNA (unpublished data). While additional cycles of PCR did produce detectable DNA for the sample generated in the absence of a primary antibody, the lack of DNA under the amplification conditions used for the microarray experiment demonstrates that nonspecific immunoprecipitation of DNA was not a confounding factor for our microarray analysis. Third, the same chromatin solution was subjected to immunoprecipitation with an anti-Mif2 antibody. Mif2 is a centromere-binding protein. Centromeres in S. cerevisiae are approximately 125 bp. The peak of Mif2 binding spanned approximately 500 bp, as assessed by PCR amplification (see Weber et al. 2004, Figure 4). This demonstrates that the shearing of fragments in the chromatin solution was sufficient to give resolution on the order of 500 bp in a case where this level of resolution is expected.

**Figure 4:**
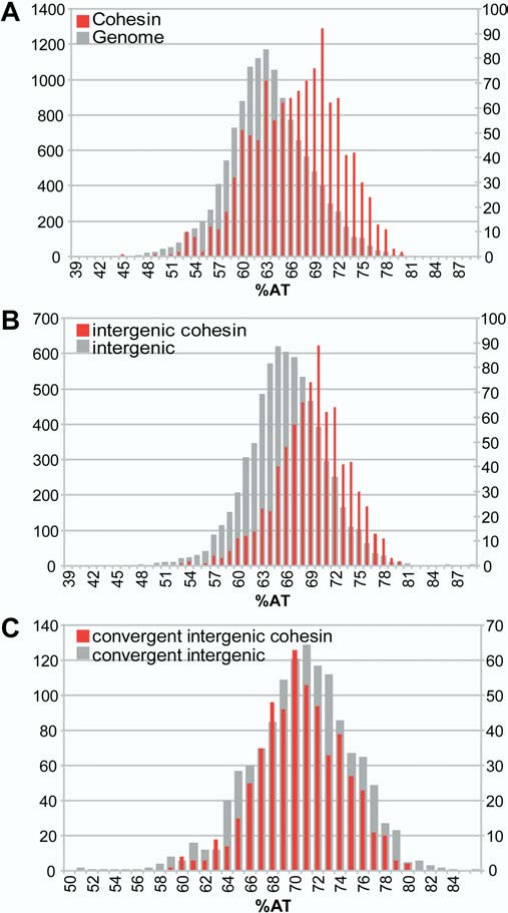
Peaks and AT Content (A) The AT content for each array element was calculated and put into bins in 1% intervals (grey bars, left y-axis). The AT content for each array element that is a cohesin peak was also put into bins (red bars, right y-axis). (B) The AT content for each intergenic array element was put into bins in 1% intervals (grey bars, left y-axis). The AT content for each intergenic array element that is a cohesin peak was also put into bins (red bars, right y-axis). (C) The AT content for each convergent intergenic array element was put into bins (grey bars, left y-axis) and the AT content for each convergent intergenic array element that is a cohesin peak was also put into bins (red bars, right y-axis).

To demonstrate the internal consistency and reproducibility of our data, we compared peaks of cohesin binding for Mcd1-18Myc in W303a (see Figure 1A), Scc1/Mcd1-6HA in A364a (Figure 1B), and Smc3-6Myc in A364a (Figure 1C). There is good agreement between the location of cohesin peaks in different strain backgrounds (correlation coefficient = 0.76 for Mcd1/Scc1 ChIP in strains A364a and W303a). This is the relevant comparison for the data in Figure 1 here and the data in Figure 1 of Weber et al. (2004), which shows the genome-wide results of ChIP for Scc1/Mcd1-6HA in the A364a background. The agreement is even stronger when different members of the cohesin complex are used as ChIP targets in the same genetic background (correlation coefficient = 0.96 for Mcd1/Scc1 and Smc3 ChIP in strain A364a). All data from individual arrays and datasets are available at http://research.stowers-institute.org/jeg/2004/cohesin/data/index.html and as supporting information (Datasets S1–S58).

### Genomic Distribution of Cohesin

**Figure 3:**
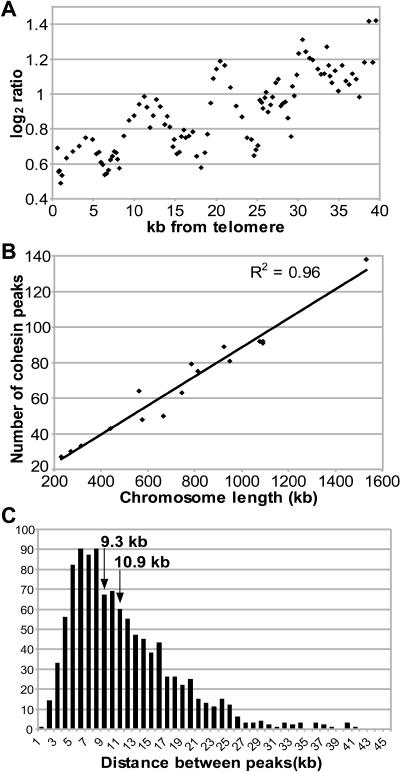
Features of Peaks (A) Using all cohesin-binding peaks within 40 kb of a telomere ordered based on distance from the telomere, we calculated a five-point moving average for distance in kilobases from the telomere (x-axis) and plotted this as a function of the five-point moving average of the log_2_ value for the associated peaks (y-axis). (B) Chromosome length (x-axis) is plotted as a function of the number of cohesin peaks (y-axis). A line was fitted using the least squares method and *R^2^* = 0.96. (C) The distance between peaks was put into 1-kb bins; the average distance between peaks is 10.9 kb and the median is 9.3 kb.

The levels of cohesin on all the chromosomes are similar and follow a clear pattern: large regions of intense binding in the pericentric domain and less intense, smaller regions distributed in a semiperiodic manner throughout the arms. We evaluated whether cohesin was associated with *cis* determinants of chromosome transmission including centromeres, telomeres, and origins of replication. Cohesin shows a large (30–50 kb), dense region of binding in pericentric domains (Figure 2). Although it has been proposed that telomeres do not associate with cohesin (Blat and Kleckner 1999), we found that nine of the 32 telomeres in fact were associated with cohesin. However, the height of the peaks associated with telomeres and subtelomeric regions is lower than at internal regions, which may reflect lower affinity or occupancy of cohesin at these regions (Figure 3A). On Chromosome III, cohesin peaks appear to be associated with replication origins that have been functionally mapped (Poloumienko et al. 2001) (see Figure 1A, only the origins with the strongest signal are indicated). Cohesin enrichment at the centromeres clearly supports previous studies implicating a requirement for the coupled function of cohesion and the centromere in chromosome segregation (Hill and Bloom 1987; Megee et al. 1999), while the significance of cohesin association with telomeres and origins is unclear.

While some cohesin is associated with the known *cis* determinants of chromosome transmission, the vast majority of sites are not. The number of cohesin-binding peaks per chromosome shows an excellent correlation to chromosome length (*R^2^* = 0.96; Figure 3B). The mean distance between sites was 10.9 kb, with a standard deviation of 6.7 kb (Figure 3C). There are 50 regions of the genome with large gaps between neighboring peaks (24 kb or greater, i.e., more than 2 s.d. from the mean); these appear to be randomly scattered throughout the arms of the larger chromosomes, and are never located on any of the four smallest chromosomes. The spacing of peaks is conserved for the most part in pericentric regions, with an additional “baseline” level of binding. Cohesin distribution therefore appears to be nonrandom with a tendency for even distribution over the genome.

### Cohesin Tends to Bind AT-Rich Sequences

Cohesin peaks were strongly associated with AT-rich regions (Figure 4). We found that 810 of the 1,095 array elements defined as cohesin-binding sites have AT content above the yeast median of 62.6% (*p* < 0.0001) (Figure 4A). Cohesin peaks are significantly associated with intergenic regions, with 765 out of 1,095, or 70%, of all peaks located in such regions (*p* < 0.0001) even though intergenic regions make up only 27% of the genome length. Intergenic regions in S. cerevisiae are more AT-rich than open reading frames (ORFs). Therefore, we tested whether the AT bias could be explained by the bias towards binding intergenic sequences by comparing the AT content of all intergenic regions with the AT content of intergenic regions associated with cohesin (598 out of 765 are above the median, *p* < 0.0001; Figure 4B). The peaks observed at ORFs are also higher in AT content than ORFs on average (*p =* 0.0005). Thus, AT content appears to be a major determinant for cohesin association.

We observed local oscillations of AT content in a 5-kb sliding window, which corresponded to cohesin-binding peaks in chromosome arms (see Figure 2), thus extending to the whole genome the observation for Chromosome III (Blat and Kleckner 1999). Furthermore, all 16 pericentric regions have local peaks of AT content (Figure 2). Interestingly, the sequence elements associated with cohesin in the pericentric domain contain nearly equal numbers of ORF and intergenic sequences, as might be expected if binding is mainly directed by the centromere and base composition and disregards other genomic features such as transcription units (Weber et al. 2004).

### Distribution of Cohesin on a YAC

**Figure 5:**
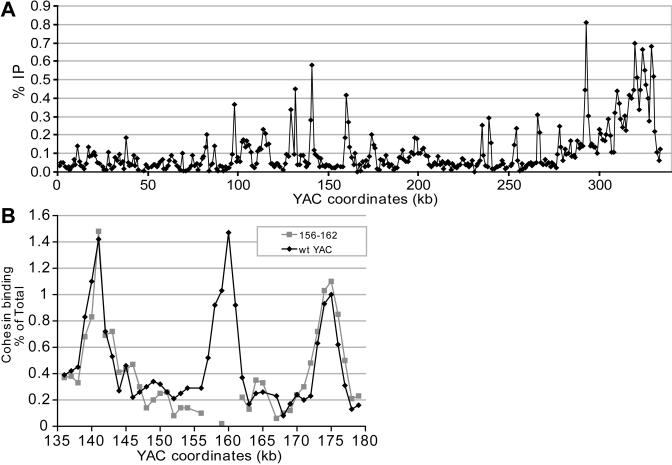
Cohesin Sites Mapped Using ChIP Followed by Semiquantitative PCR with Primers at 1-kb Intervals in a YAC Containing Human DNA (A) Cohesin binding for the entire YAC is shown. (B) Cohesin binding in the region spanning 135–180 kb is shown for the wild-type YAC (black diamonds) and for the YAC containing a replacement of the sequences at 156–162 kb with the gene encoding geneticin resistance (grey squares).

The semiregular spacing of cohesin and the correlation with local oscillations of base composition suggested that AT content and/or a measuring mechanism might control cohesin distribution on the chromosome. In order to test these possibilities, we used a nonessential ectopic yeast artificial chromosome (YAC). We used ChIP followed by quantitative PCR to map cohesin-binding sites in a YAC containing 334 kb of human DNA from Chromosome VII. The pericentric region on the right end of the YAC shows a broad (approximately 35 kb), intense association with cohesin. This is similar to the cohesin association with endogenous pericentric regions. However, the spacing of cohesin does not have the same periodic nature as on endogenous chromosomes. For example, the leftmost 83 kb of the YAC contains only one peak of cohesin binding, resulting in two large gaps for cohesin of 38 and 45 kb (Figure 5A). These two gaps are larger than any of the gaps found on the smaller endogenous yeast chromosomes, which have a comparable size to the YAC. The human DNA fragment does not contain oscillations of AT content similar to those observed in the yeast genome, nor does the pattern of cohesin association appear to reflect base composition in this context. Thus, the difference in the distribution of cohesin-binding sites in the arms of the YAC supports the idea that sequence contributes to cohesin distribution in yeast and that evolution has selected for an even distribution on endogenous chromosomes.

To further test the contribution of sequence to cohesin binding, we took advantage of the fact that none of the human sequences were essential for yeast survival. When we replaced the region from 156 to 162 kb, which contains a cohesin-binding site, with the gene encoding for geneticin resistance, this region was no longer associated with cohesin (Figure 5B). Neighboring regions were unaffected, and de novo cohesin binding was not observed. This suggested that some property of the sequence, rather than its precise location or context, was responsible for cohesin binding.

### Transcription and Cohesin

Inspection of the intergenic regions associated with cohesin revealed a strong preference towards the intergenic regions in which transcription is converging, and additionally, an extreme bias against association with intergenic regions in which transcription is diverging. Among the cohesin-associated intergenic regions that could be assigned to a category, 86% were in intergenic regions with converging transcription, 12% were in intergenic regions with surrounding unidirectional transcription, and only 2% were in intergenic regions that are between two divergently transcribed genes. In contrast, the genome as a whole has these regions in approximately a 1:2:1 ratio, respectively, making this result highly statistically significant (*p <* 0.0001). In fact, 39% of the convergent intergenic regions in the genome have peaks of cohesin binding, and nearly half of all cohesin-binding sites are in convergent intergenic regions. These percentages approach the predictive power of consensus sequences for identifying binding sites of their cognate transcription factor (Chu et al. 1998; Lieb et al. 2001). Convergent intergenic regions have high AT content compared to the genome at large; the bias of the intergenic sequences associated with cohesin can be partly explained by the AT bias of convergent intergenic regions (see Figure 4C).

Of the sites, 865 of 1,095 can be explained by one or more of the following three factors: (1) location within 25 kb of a centromere, (2) above average AT content, or (3) location in an intergenic region with converging transcription. This leaves 230 sites unexplained. Of these, 43 are intergenic. Most of these are simply difficult to assign to a transcriptional category. Interestingly, of the 230 “unexplained” sequences, 187 are in ORFs, which is more than half of all the ORFs associated with cohesin. These ORFs do not appear to have any unifying theme with regard to function, dubiousness, transcription level, or essentiality. The 187 peaks have similar height to other peaks. Thus, unlike the *cis* determinants in the intergenic regions, the factors within the ORFs that enable cohesin binding are not well understood.

Attempts to identify a genome-wide linear consensus binding site for cohesin using BioProspector (Liu et al. 2002), MobyDick (Bussemaker et al. 2000), AlignACE (Hughes et al. 2000), and MEME (Bailey and Elkan 1994) did not return any sequence model with predictive value. However, when we took the group of “unexplained” ORF sequences and looked for a common motif using BioProspector, we identified two repetitive sequences that were strongly enriched relative to the genome: (CAR)_5_ (*p =* 10^−65^) and (GAN)_10_ (*p =* 10^−76^). The *p* values reflect the significance of these sequences as calculated based on a Monte Carlo simulation for the yeast genome. These sequences were rare in intergenic regions in yeast. These sequences were not present in the YAC. Further experiments will be required to determine whether these repeats are targeted by cohesin. Interestingly, human cohesin has been localized in Alu repeats (Hakimi et al. 2002), suggesting that repetitive DNA may be prone to a particular structure or chromatin configuration preferred by cohesin, or may accumulate in regions that are bound by cohesin. Alu repeats do not bear any obvious similarity to the sequences we identified. Binding of cohesin may help modulate transcription of these repeated sequences.

### The Mechanism of the Negative Association between Transcription and Cohesin Binding

The link between intergenic tail-to-tail regions and cohesin suggests a general incompatibility between transcription and cohesin association**.** The observations that transcription can disrupt centromeric cohesin and results in chromosome missegregation and cell death (Tanaka et al. 1999), and that cohesin binds at the boundaries of silent chromatin in several loci (Laloraya et al. 2000) are in agreement with this.

**Figure 6:**
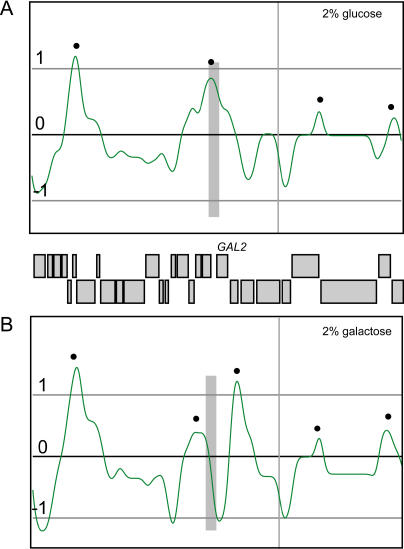
Transcription Affects the Cohesin Peak at the Promoter of *GAL2* SGD coordinates 260–320 kb (x-axis) and a gene map are depicted for Chromosome XII. The strain 1827-22D (isogenic to the strain in Figure 1 except *CDC16*) was grown with either 2% glucose (A) or 2% galactose (B) as the carbon source. Cultures were arrested with nocodazole, and ChIP chip was performed. The smoothed data (as the log_2_ of the ratio) is depicted in green, the peaks found by PeakFinder are indicated with black dots, and the region corresponding to the *GAL2* promoter is indicated with a grey bar. Transcription of *GAL2* is up-regulated 42-fold in (B).

We analyzed whether changing the transcriptional program could change the association of cohesin with a locus. We grew cultures with either 2% glucose or 2% galactose as the carbon source and arrested them in metaphase using nocodazole. The main difference between metaphase arrest in the presence of nocodazole and in *cdc16*-ts is that cohesin binding at pericentric regions is increased in the former case (unpublished data). We carried out ChIP chip analysis in parallel with monitoring gene expression in the same cells using ORF arrays. Of the regions where gene expression changed 5-fold or more, only two regions were associated with cohesin. One peak of cohesin binding in glucose was associated with the promoter of the *GAL2* gene (Figure 6A), which was induced 42-fold in galactose. This had a dramatic effect on the local profile of cohesin binding (Figure 6B). The promoter region of *GAL2* became a trough of cohesin binding, and the single peak observed in glucose was split into two peaks, in effect adding a new peak to the region. The second region was an uncharacterized ORF, YDL218W, a membrane protein distantly related to secretory factor *NCE102*/YPR149W and to metazoan synaptogyrin family (unpublished data). Expression of YDL218W was induced 11-fold in the presence of glucose compared to galactose. This ORF is associated with cohesin in the presence of galactose, but this association is reduced when glucose is present (unpublished data). The peaks surrounding both regions were unaffected. These results demonstrate that high levels of transcription are incompatible with cohesin binding. It also supports the results observed with the human DNA that neighboring cohesin sites behave independently.

**Figure 7:**
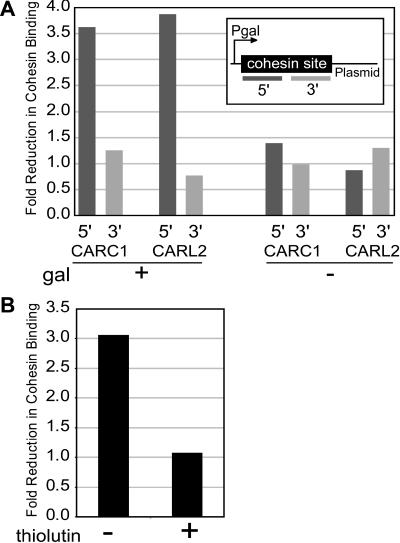
Effect of Transcript Elongation on Cohesin Associated with CARC1 and CARL2 Located on a Plasmid Next to a Galactose-Inducible Promoter (A) The fold reduction in cohesin binding in the presence (+) or absence (−) of galactose-induced transcription is depicted as a function of the 5′ or 3′ end of the locus. (B) The fold reduction in cohesin binding at CARL2 during galactose-induced transcription in the presence (+) or absence (−) of thiolutin, an inhibitor of transcript elongation.

There are a number of mechanisms that could account for the incompatibility between transcription and cohesin binding. Transcription of a region during G1 may prevent new association of cohesin, or transcription may displace cohesin in G2. We explored the mechanistic link between transcription and cohesin association at the previously characterized cohesin sites cohesin-associated region on Chromosome III (C) (CARC1) and cohesin-associated region on Chromosome XII (L) (CARL2) (Laloraya et al. 2000) by inserting 0.8 kb of CARC1 of 1.4 kb of CARL2 into a plasmid construct next to a galactose-inducible promoter (pGAL1-10). Strains containing one of these two plasmids were grown with 2% raffinose as the carbon source, arrested in G1 with alpha factor, and then released from G1 in the presence of nocodazole, producing a metaphase arrest. Galactose was added to half the culture. One hour after the addition of galactose, cultures were fixed with formaldehyde and processed for ChIP. Semiquantitative PCR was used to monitor the distribution of cohesin. With the appropriate use of primers, cohesin association with CARC1 and CARL2 on the plasmid and the endogenous locus could be distinguished. Galactose-induced transcription had no effect on association of cohesin with the endogenous loci (unpublished data) but disrupted cohesin associated with the 5′ end of both CARC1 and CARL2 on the plasmid (Figure 7A). This result demonstrates that transcription during G2 can displace cohesin.

The displacement of cohesin may be due to a competition between RNA pol II/chromatin–remodeling factors and cohesin for DNA, or transcript elongation may remove cohesin. We tested if the incompatibility was dependent on transcript elongation. A culture was arrested with nocodazole, and galactose-responsive transcription was induced by the addition of galactose, as described above. Thiolutin was added to half of this culture immediately prior to the addition of galactose. Thiolutin inhibits transcript elongation but presumably does not inhibit the binding of RNA pol II (Parker et al. 1991). The effect as monitored at CARL2 was dependent upon elongation since the addition of thiolutin prevented the displacement of cohesin (Figure 7B). This result demonstrates that the binding of RNA pol II per se does not affect cohesin binding, but transcript elongation can displace cohesin within the G2 portion of a single cell cycle.

### Meiotic Cohesin

**Figure 8:**
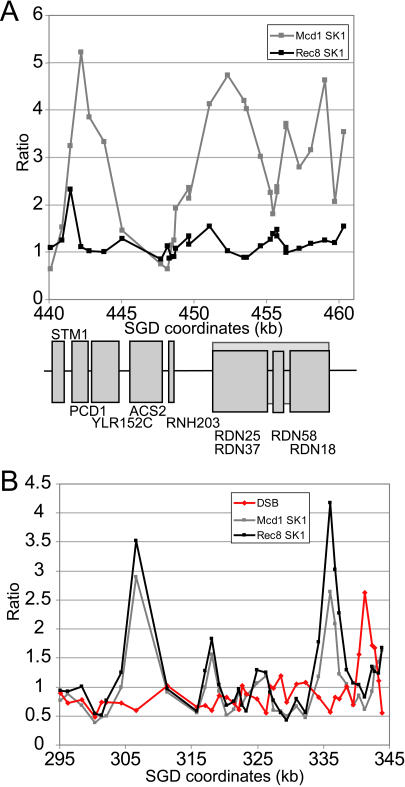
Meiotic Cohesin DSB data are shown in red, Rec8 data in black, and Mcd1 data in grey. (A) Ratios for meiotic cohesin are compared to mitotic cohesin in SK1 for kilobasepairs 440–461 on Chromosome XII. For the mitotic culture, cells were arrested with nocodazole. For meiotic cells, timepoints were collected every 2 h from hour 4 to hour 12 after transfer to SPM. The median ratio value was used to represent the data. Meiosis is slower in an SK1 strain with an HA-epitope-tagged Rec8 than in a wild-type strain (see Figure 9). The gene structure for this locus is shown below the graph, with genes encoded by the Watson strand labeled on top and genes encoded by the Crick strand labeled on the bottom. (B) Ratios for meiotic cohesin are compared to mitotic cohesin and DSBs for kilobasepairs 295–345 on Chromosome XII.

**Figure 9:**
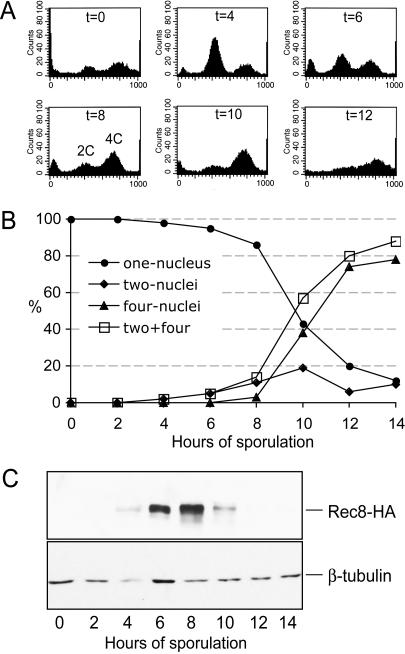
Meiotic Timecourse for an SK1 Strain Containing Rec8-3HA Cells were collected at the indicated timepoints throughout meiosis using the same experimental regime used to collect the binding sites of Rec8-3HA presented in Figure 8. The epitope tag appears to slow meiosis by 3–4 h as compared to an untagged strain. Three assays were developed to monitor culture synchrony during meiosis. (A) FACS profile of the *REC8-3HA* strain. Aliquots of cells were fixed with 70% EtOH, followed by FACS analysis. (B) Nuclear division of the *REC8-3HA* strain. Aliquots of cells were fixed with 1% formaldehyde for 1 h at room temperature. Nuclear DNA was stained by DAPI and visualized under a fluorescence microscope. At least 200 cells were scored at each timepoint. (C) Rec8-3HA protein level. Protein extracts were prepared and subjected to SDS-PAGE and Western blot. The Rec8-3HA protein level was detected by an anti-HA antibody (12CA5). The same blot was stripped and reprobed with anti-β-tubulin antibody to detect the level of β-tubulin, which served as a loading control.

The transcriptional program of a cell changes under different conditions, such as the developmental program of sporulation. We used ChIP chip to analyze the location of the meiosis-specific cohesin complex (Klein et al. 1999; Watanabe and Nurse 1999). The protein composition of the meiosis-specific complex has been described, but no information on the *cis* determinants of this complex has been reported. We expressed Rec8-3HA in SK1, a rapidly and synchronously sporulating strain, and analyzed the location of the cohesin–DNA complex in ChIP experiments (Figure 8). The pattern of cohesin association in meiotic and mitotic cells appears to be similar (correlation coefficient = 0.77 across SK1 genome comparing Rec8 to Mcd1; see Figure 8 for coordinates 295–345 kb and 440–460 kb on Chromosome XII; additional data regarding the timecourse of sporulation is provided in Figure 9). In both cases, pericentric regions contain broad, intense regions of cohesin association and there are nonrandomly spaced cohesin sites in the arms.

*PCD1* has been shown to be a cohesin-binding site (Laloraya et al. 2000). Binding to this site is diminished for meiotic cohesin (see Figure 8A). The transcription of this gene is induced early in meiosis, which may explain why cohesin binding to this site is diminished (Chu et al. 1998). Other genes show a similar pattern, namely that they are binding sites for cohesin in mitotic cells, but their transcription is induced early in meiosis, and they do not appear to be binding sites for the Rec8-containing cohesin complex (e.g., *YPR006C, YDL238C,* and *YER179W*). This suggests that binding of the meiotic complex, like the mitotic complex, is not compatible with transcription.

We compared the location of meiotic cohesin to the location of the double-strand breaks (DSBs) that initiate meiotic recombination (Gerton et al. 2000). We found a negative correlation (correlation coefficient = −0.26); DSBs tend to occur in regions where meiotic cohesin is absent, and meiotic cohesin tends to be located in regions that contain low levels of DSBs (see Figure 8B). Cohesin has been shown to be required for the formation of the axial elements that become the lateral elements of the proteinaceous structure known as the synaptonemal complex (SC) (Klein et al. 1999). The SC organizes meiotic chromosomes and aids interhomolog recombination. In fact, meiotic cohesin has been shown to be required for meiotic recombination (Klein et al. 1999). This result suggests that recombination proteins can recognize chromosome structure/organization provided by cohesin.

The most notable difference between meiotic and mitotic cohesin is at the ribosomal DNA (rDNA) locus. (Nocodazole arrest does not affect cohesin binding in the rDNA in A364a or W303a, unpublished data) The rDNA is encoded in an approximately 1–2-Mb region on the right arm of Chromosome XII consisting of 100–200 tandem copies of a 9.1-kb repeat. There is a peak of cohesin binding that localizes to the left border of the rDNA repeat (Laloraya et al. 2000) that is absent for meiotic cohesin (see Figure 8A). Although information regarding the transcription of rDNA in meiosis is unavailable, genes involved in the processing of the 35S transcript, such as *ROK1, RRS1*, and *EBP2* (Wade et al. 2001), are down-regulated 5- to 15-fold by 0.5 h in meiosis, and ribosomal protein genes are also down-regulated an average of 5-fold (Chu et al. 1998), suggesting that transcription of this region is significantly reduced in meiosis. The rDNA is located in the nucleolus in meiotic cells and is associated with proteins that repress interhomolog recombination (Petes and Botstein 1977; San-Segundo and Roeder 1999, 2000). This region may have a chromatin structure in meiosis that suppresses transcription, recombination (so as to avoid chaotic exchange between repeated elements), and cohesin binding.

## Discussion

We have used ChIP chip to map, to 1–2-kb resolution, the genome-wide pattern of cohesin association under several different growth conditions (metaphase arrest by *cdc16*-ts or nocodazole, galactose versus glucose as a carbon source, and induction of meiosis) and in three different yeast-strain backgrounds (W303a, SK1, and A364a). Using PeakFinder, a program that assesses cohesin binding by comparison of signal to variable local background, we find that the majority of cohesin-binding sites are remarkably constant under these different circumstances. Distribution of cohesins throughout the genome appears to depend on a combination of base composition, sequence, and transcriptional activity. We find evidence for three types of cohesin sites in the genome: (1) the centromere and pericentric domain, (2) intergenic regions in chromosome arms, and (3) ORFs in chromosome arms. The association of cohesin with these three types of sites is subject to different genomic parameters. Cohesin at centromeres and pericentric regions is spread over a broad domain with an elevated “baseline” level and is not affected by the natural transcriptional and coding status. Much of the cohesin in chromosome arms is located in transcriptionally converging intergenic regions. ORFs in chromosome arms where cohesin is found are enriched for repetitive sequences. This suggests that there may be three mechanisms to load cohesin, consistent with what has been proposed for cohesin in meiotic chromosomes for S. pombe (Kitajima et al. 2003). A unifying feature of all three types of sites is high AT content.

Pericentric regions contain the most intense and broadest levels of cohesin in the genome (for a more complete analysis of pericentric cohesin see Weber et al. [2004]). This finding is consistent with a model in which a centromere contains determinants of two opposing processes: (1) pulling the chromosomes apart, via the assembled kinetochore attached to a microtubule, and (2) keeping chromosomes together, via pericentric cohesion. The intensity and breadth of cohesin binding at pericentric regions is similar for all chromosomes, implying microtubules pull all chromosomes with comparable force, regardless of their length. On the other hand, the number of binding sites per chromosome is proportional to chromosome length. This result implies that arm cohesion is not a direct measure of the force exerted by spindle microtubules, and may serve a different function, for instance, to achieve similar levels of condensation. The model in budding yeast that cohesin can participate in genome maintenance in two ways, namely condensation via arm cohesin and biorientation via pericentric cohesin, is intriguing in light of the recent finding that cohesin complexes with different subunits are found on arms and pericentric regions on meiotic chromosomes in S. pombe and apparently serve different functions (Kitajima et al. 2003).

Cohesin cannot stay bound to DNA in the face of active transcript elongation based on three independent cohesin sites (promoter of *GAL2,* CARC1, and CARL2). If cohesin and transcript elongation were incompatible, then we would also expect to find sites biased towards intergenic regions, which we do. However, we find a strong bias towards intergenic regions with converging transcription, and a bias against intergenic regions with surrounding unidirectional transcription or diverging transcription, suggesting that intergenic regions with converging transcription may have especially low transcription. These regions may have evolved particularly strong transcriptional stops since they are quite short on average and the cell may need to avoid transcription from one side extending to the other to prevent the synthesis of antisense RNA. The protection of sequence elements important for the replication and segregation of eukaryotic chromosomes from transcription may be a general necessity for their proper function in vivo. For instance, transcription through an autonomous replicating sequence (Snyder et al. 1988) or a centromere (Hill and Bloom 1987) disrupts their function.

The observed antagonistic relationship between transcription and cohesin binding in chromosome arms can be explained in two ways. Firstly, transcript elongation may be directly responsible for displacing cohesin. In this type of model, cohesin loading/binding is random, and transcription (and possibly other DNA metabolic processes) “pushes” cohesin into place or strips cohesin from inappropriate locations in each cell cycle. Secondly, transcript elongation may be indirectly responsible for localizing cohesins, for example by accumulation of “nonpermissive” chromatin in transcribed regions and “permissive” chromatin in nontranscribed regions. This type of genome-wide demarcation of transcription units has been shown to occur in S. cerevisiae (Nagy et al. 2003) and may depend on nucleosomes (Lee 2004) and histone variants. The chromatin remodeling complex RSC (Remodels the Structure of Chromatin) has recently been shown to be important for establishment of cohesin in chromosome arms (Baetz et al. 2004; Huang et al. 2004). The preferential location of cohesin in heterochromatin in S. pombe also supports the idea of chromatin modification/structure as the basis for cohesin localization (Bernard et al. 2001; Nonaka et al. 2002). The possibility also exists that cohesin itself may influence transcriptional status and act as a transcriptional boundary (Hagstrom and Meyer 2003; Rollins et al. 1999).

Despite the subunit difference between the meiotic and mitotic cohesin complex, we find that the association of cohesin with DNA in meiotic cells is similar to that in mitotic cells. In addition, we find that the constitutive peaks of meiotic cohesin binding are negatively correlated with DSB sites. This negative correlation is consistent with the model proposed by Blat et al. (2002) for the relationship between recombination and cohesin. This model suggests that cohesins are at meiotic chromosome cores and that recombination occurs in chromatin loops emanating from these cores where the part of the loop undergoing recombination is transiently localized to the axis. Thus, the recombination machinery can sense chromosome organization provided by cohesin. The differences in binding of meiotic and mitotic cohesin suggest that the location of the meiotic complex is also dependent on gene transcription. Hence, meiotic recombination in a given organism may be somewhat dependent on the spacing of cohesin as established in premeiotic S phase, which is in turn responsive to transcription. The genome-wide distribution of DSBs is positively correlated with regions of high GC content, divergent promoters, and transcription factor binding (Gerton et al. 2000). Thus transcription, recombination, and cohesion all display intimate connections to chromosome and chromatin structure.

Genome-wide studies of protein–DNA complexes afford a better understanding of the role of these complexes in the biology of an organism and its genome. In the process of analyzing the first genome-wide map of cohesin in any organism, we developed PeakFinder, a program able to sensitively identify binding sites of protein–DNA complexes in their local genomic environment, and potentially useful for analysis of any other genome-wide measurements. While budding yeast appears to have largely opted for placement of cohesin in AT-rich, transcriptionally inactive regions, other organisms with much longer and more complicated transcriptional units, different base composition properties, or different levels of condensation may employ different strategies for the placement of cohesin, which may in turn affect the stability of those genomes. The genome-wide analysis of cohesin in S. cerevisiae will serve as a useful framework upon which to explore attributes of cohesin localization in higher eukaryotes.

## Materials and Methods

### 

#### ChIP methods.

ChIPs were performed as previously described (Meluh and Koshland 1997; Laloraya et al. 2000). Semiquantitative PCR analysis was performed as previously described (Laloraya et al. 2000). ChIP using the same experimental regime in a strain lacking the Myc epitope was performed and did not yield any appreciable signal (Megee et al. 1999).

#### Cell culture.

For the meiotic timecourse, cultures were grown in YPA, then transferred to SPM. Timepoints were removed for ChIP at 4, 6, 8, 10, and 12 h after transfer to SPM. Nocodazole-mediated arrest was accomplished by adding nocodazole to a final concentration of 15 μg/ml to the media. All cultures were grown at 30 °C. Shifting cultures to 37 °C in prewarmed media induced metaphase arrest in *cdc16*-ts cells.

#### DNA amplification, labeling, and hybridization.

Preparation of Cy5- and Cy3-labeled DNA, hybridization, and analysis were performed as previously described (Bohlander et al. 1992; Gerton et al. 2000). The polyL-lysine-coated spotted glass microarrays used in this study contained each ORF and each intergenic region in the yeast genome as individual spots (Iyer et al. 2001). For each experimental condition, a minimum of two independent immunoprecipitations was performed. DNA from the immunoprecipitation was labeled with Cy5 and competitively hybridized with total genomic DNA labeled with Cy3. Hybridizations with fluor reversal were also performed for DNA from at least one of the immunoprecipitations for each condition. At least three arrays were analyzed per experimental condition, and the median values were used to represent the dataset. Hybridizations were performed at 63 °C overnight under standard conditions, and slides were washed successively with 0.6X SSC/0.03% SDS and then 0.06X SSC prior to scanning (see also http://microarrays.org). The meiotic experiments were done in the SK1 strain background and although two independent timecourses were performed, the results from a single representative timecourse were used for analysis. The resolution of these genome-wide maps is limited by (1) the shear size of the DNA fragments (range of 200–1000 bp) and (2) the size of the elements on our microarrays (mean of 0.9 kb). We do not expect these fragment-size distributions to introduce a significant bias in our mapping effort since the previously estimated size of a cohesin-binding region at an arm site is 0.8–1.0 kb (Laloraya et al. 2000).

#### Computational methods.

The arrays were scanned using an Axon Instruments (Union City, California, United States) 4000B scanner and quantitated using GenePix 4.0. Results were stored in the AMAD database. Data were normalized and filtered by requiring intensity to be 200 or more, and spots to have a correlation coefficient of 0.5 or more. For analysis purposes, any feature with less than two measurements was excluded (with the exception of the meiotic timecourse). Data were analyzed using PeakFinder, a program developed specifically for finding peaks in ChIP data, but generally applicable for plotting any measurement against genomic coordinates, smoothing the curves, and annotating peaks on the basis of local properties of the curve. Extensive documentation for PeakFinder is available at http://research.stowers-institute.org/jeg/2004/cohesin/peakfinder/. Briefly, PeakFinder takes the fluorescence ratios and samples them at the indicated interval of basepairs. The log_2_ of the data are then smoothed. The first derivative of the smoothed line is used to identify peaks, and the absolute value of the corresponding peak is then extracted from the raw data (this is necessary because the nature of the smoothing algorithm dampens the peak height). PeakFinder allows filtering of peaks based on the parameters of the peak. For example, cohesin peaks analyzed in the *cdc16*-ts dataset were identified using the following set of parameters: (1) sampling log_2_-transformed ratios at 100-bp intervals, (2) smoothing over eight rounds using a nine-point Gaussian-weighted moving average, and (3) filtering of peaks with a left and right rise of less than 0.1 and a height less than 0.4 (log_2_ space). These conditions identified all peaks mapped to high resolution on Chromosomes III and XII (Laloraya et al. 2000). A current limitation of PeakFinder is that it is unable to identify one-sided peaks; therefore telomeres were manually inspected for cohesin binding. PeakFinder is written in Delphi, runs on a Windows platform, and is distributed under the GNU General Public License.

#### YAC.

PCR primers were designed to amplify 150–300-bp sequences, at 1-kb intervals along the entire length of the 1572 YAC (Green et al. 1995). Nucleotides 1–3,683 contain the vector sequences from pYAC4 including the telomere and *URA3*. Nucleotides 326,702—332,707 contain vector sequences from pYAC4 including the centromere, *TRP1,* and *ARS1*. The YAC was introduced into the strain 1377 A1 4B, two independent cultures were grown to exponential phase, and nocodazole was added. After 3 h of growth at 23 °C, more than 90% of cells were arrested in metaphase. Cultures were processed for ChIP as described previously.

#### Thiolutin.

0.8 kb from CARC1 and 1.4 kb from CARL2 were cloned into pUNI and then recombined with pYCE to form pCM34 and pCM38. This places the pGAL1-10 promoter immediately adjacent to cohesin-associated regions. pCM34 and pCM38 were introduced into 1377 A1 4B by transformation. Strains with pCM34 and pCM38 were initially grown in complete medium lacking uracil with raffinose as a carbon source. This medium selects for retention of the plasmids and prevents transcription from the Gal-inducible promoter. These cultures were diluted approximately 100-fold in YEP raffinose and grown to 7 × 10^6^/ml. Cultures were arrested in G1 with alpha factor, released from G1 in the presence of nocodazole, and grown for 3 h to generate an M phase arrest. Cultures were split in two, and one half received galactose to a final concentration of 4%. One hour after addition of galactose, cultures were fixed and processed for ChIP. Experiments with thiolutin were performed as described above except thiolutin was added to a final concentration of 3 μg/ml just prior to galactose addition. Primers were generated that amplified 5′ and 3′ regions of CARC1 and CARL2 in the endogenous locus and on pCM34 and pCM38. Reduction in cohesin binding was expressed as the ratio of (1) the amount of cohesin bound to the 5′ or 3′ ends of the CAR on the plasmids to (2) the amount of cohesin bound to the 5′ or 3′ regions of the CAR in the genome.

## Supporting Information

Datasets S10–S58 correspond to the individual GenePix results (GPR) files for each array performed. For each dataset we have listed the Cy3 channel sample and the Cy5 channel sample.

Dataset S1 W303 Strain Arrested by *cdc16*-ts with ChIP Performed for Mcd1-18Myc File cdc16_Mcd1-18Myc_W303.
(483 KB TXT).

Dataset S2 A364a Strain Arrested by *cdc16*-ts with ChIP Performed for Mcd1-6HA File cdc16_Mcd1-6HA_A364a.
(957 KB TXT).

Dataset S3 A364a Strain Arrested by *cdc16*-ts with ChIP Performed for Smc3-6Myc File cdc16_Smc3-6Myc_A364a.
(701 KB TXT).

Dataset S4 W303 Strain Grown in Galactose and Arrested by Nocodazole with ChIP Performed for Mcd1-18Myc File Mcd1_18Myc_W303_NZgalCHIP.
(406 KB TXT).

Dataset S5 W303 Strain with Mcd1-18Myc Grown in Galactose and Arrested by Nocodazole with RNA Harvested for Gene Expression File Mcd1-18Myc_W303_NZgal_exp.
(196 KB TXT).

Dataset S6 W303 Strain with Mcd1-18Myc Grown in Glucose and Arrested by Nocodazole with RNA Harvested for Gene Expression File Mcd1-18Myc_W303_NZglu_exp.
(221 KB TXT).

Dataset S7 W303 Strain Grown in Glucose and Arrested with Nocodazole with ChIP Performed for Mcd1-18Myc File Mcd1-18Myc_W303_NZgluChIP.
(694 KB TXT).

Dataset S8 SK1 Strain Arrested with Nocodazole with ChIP Performed for Mcd1-3HA File Mcd1-3HA_SK1_NZ.
(339 KB TXT).

Dataset S9 SK1 Strain in Meiosis with ChIP Performed for Rec8-3HA File Rec8-3HA_SK1.
(710 KB TXT).

Dataset S10 SIMRUP2_147 Cy3 = ChIP *cdc16*-ts Mcd1-6HA in A364a; Cy5 = genomic DNA.
(4.6 MB XLS).

Dataset S11 SIMRUP2_170 Cy3 = ChIP *cdc16*-ts Mcd1-6HA in A364a; Cy5 = genomic DNA.
(4.6 MB XLS).

Dataset S12 SIMRUP2_171 Cy3 = ChIP *cdc16*-ts Mcd1-6HA in A364a; Cy5 = genomic DNA.
(4.6 MB XLS).

Dataset S13 SIMRUP2_178 Cy3 = genomic DNA; Cy5 = ChIP *cdc16*-ts Mcd1-6HA in A364a.
(4.6 MB XLS).

Dataset S14 SIMRUP2_180 Cy3 = genomic DNA; Cy5 = ChIP *cdc16*-ts Mcd1-6HA in A364a.
(4.6 MB XLS).

Dataset S15 SIMRUP2_187 Cy3 = ChIP *cdc16*-ts Smc3-6Myc in A364a; Cy5 = genomic DNA.
(4.6 MB XLS).

Dataset S16 SIMRUP2_190 Cy3 = ChIP *cdc16*-ts Smc3-6Myc Mcd1-6HA ChIP for HA in A364a; Cy5 = genomic DNA.
(4.6 MB XLS).

Dataset S17 SIMRUP2_191 Cy3 = ChIP *cdc16*-ts Smc3-6Myc Mcd1-6HA ChIP for Myc in A364a; Cy5 = genomic DNA.
(4.6 MB XLS).

Dataset S18 SIMRUP2_226 Cy3 = genomic DNA; Cy5 = ChIP *cdc16*-ts Smc3-6Myc in A364a.
(4.6 MB XLS).

Dataset S19 SIMRUP2_244 Cy3 = genomic DNA; Cy5 = ChIP *cdc16*-ts Smc3-6Myc Mcd1-6HA ChIP for HA in A364a.
(4.6 MB XLS).

Dataset S20 SIMRUP2_254 Cy3 = genomic DNA; Cy5 = ChIP *cdc16*-ts Smc3-6Myc Mcd1-6HA ChIP for Myc in A364a.
(4.6 MB XLS).

Dataset S21 UP2_13 Cy3 = genomic DNA; Cy5 = ChIP *cdc16*-ts Mcd1-18Myc in W303.
(2.5 MB XLS).

Dataset S22 UP2_19 Cy3 = genomic DNA; Cy5 = ChIP *cdc16*-ts Mcd1-18Myc in W303.
(2.5 MB XLS).

Dataset S23 UP3_186 Cy3 = ChIP 6h Rec8-3HA in SK1; Cy5 = genomic DNA.
(2.6 MB XLS).

Dataset S24 UP3_187 Cy3 = ChIP 8h Rec8-3HA in SK1; Cy5 = genomic DNA.
(2.5 MB XLS).

Dataset S25 UP3_188 Cy3 = ChIP 10h Rec8-3HA in SK1; Cy5 = genomic DNA.
(2.6 MB XLS).

Dataset S26 UP3_190 Cy3 = ChIP 6h Rec8-3HA in SK1; Cy5 = genomic DNA.
(2.6 MB XLS).

Dataset S27 UP3_191 Cy3 = ChIP 8h Rec8-3HA in SK1; Cy5 = genomic DNA.
(2.6 MB XLS).

Dataset S28 UP3_29 Cy3 = genomic DNA; Cy5 = ChIP 12h Rec8-3HA in SK1.
(2.6 MB XLS).

Dataset S29 UP3_30 Cy3 = genomic DNA; Cy5 = ChIP 4h Rec8-3HA in SK1.
(2.6 MB XLS).

Dataset S30 UP3_48 Cy3 = genomic DNA; Cy5 = ChIP *cdc16*-ts Mcd1-18Myc in W303.
(2.6 MB XLS).

Dataset S31 UP3_51 Cy3 = genomic DNA; Cy5 = ChIP *cdc16*-ts Mcd1-18Myc in W303.
(2.6 MB XLS).

Dataset S32 UP3_84 Cy3 = ChIP *cdc16*-ts Mcd1-18Myc in W303; Cy5 = genomic DNA.
(2.6 MB XLS).

Dataset S33 UP3_85 Cy3 = ChIP *cdc16*-ts Mcd1-18Myc in W303; Cy5 = genomic DNA.
(2.6 MB XLS).

Dataset S34 UP3_86 Cy3 = genomic DNA; Cy5 = ChIP 4h Rec8-3HA in SK1.
(2.6 MB XLS).

Dataset S35 UP3_87 Cy3 = genomic DNA; Cy5 = ChIP 10h Rec8-3HA in SK1.
(2.6 MB XLS).

Dataset S36 UP3_89 Cy3 = genomic DNA; Cy5 = ChIP 12h Rec8-3HA in SK1.
(5.6 MB XLS).

Dataset S37 UP4_224 Cy3 = genomic DNA; Cy5 = ChIP nocodazole arrest Mcd1-3HA in SK1.
(2.6 MB XLS).

Dataset S38 UP4_225 Cy3 = genomic DNA; Cy5 = ChIP nocodazole arrest Mcd1-3HA in SK1.
(2.6 MB XLS).

Dataset S39 UP5_164 Cy3 = genomic DNA; Cy5 = ChIP nocodazole arrest glucose Mcd1-18Myc in W303.
(2.7 MB XLS).

Dataset S40 UP5_80 Cy3 = genomic DNA; Cy5 = ChIP nocodazole arrest glucose Mcd1-18Myc in W303.
(2.7 MB XLS).

Dataset S41 UP6_124 Cy3 = ChIP nocodazole arrest glucose Mcd1-18Myc in W303; Cy5 = genomic DNA.
(2.6 MB XLS).

Dataset S42 UP6_210 Cy3 = genomic DNA; Cy5 = ChIP nocodazole arrest glucose Mcd1-18Myc in W303.
(4.4 MB XLS).

Dataset S43 UP6_213 Cy3 = genomic DNA; Cy5 = ChIP nocodazole arrest glucose Mcd1-18Myc in W303.
(4.4 MB XLS).

Dataset S44 UP6_214 Cy3 = genomic DNA; Cy5 = ChIP nocodazole arrest galactose Mcd1-18Myc in W303.
(4.4 MB XLS).

Dataset S45 UP6_217 Cy3 = ChIP nocodazole arrest glucose Mcd1-18Myc in W303; Cy5 = genomic DNA.
(4.4 MB XLS).

Dataset S46 UP6_218 Cy3 = ChIP nocodazole arrest galactose Mcd1-18Myc in W303; Cy5 = genomic DNA.
(4.4 MB XLS).

Dataset S47 UP6_221 Cy3 = ChIP nocodazole arrest galactose Mcd1-18Myc in W303; Cy5 = genomic DNA.
(4.4 MB XLS).

Dataset S48 UP6_223 Cy3 = genomic DNA; Cy5 = ChIP nocodazole arrest glucose Mcd1-18Myc in W303.
(4.4 MB XLS).

Dataset S49 UP6_225 Cy3 = genomic DNA; Cy5 = ChIP nocodazole arrest galactose Mcd1-18Myc in W303.
(4.4 MB XLS).

Dataset S50 YA1S4P2_106 Cy3 = polyA+ reference RNA; Cy5 = polyA+ RNA nocodazole arrest galactose Mcd1-18Myc in W303.
(2.2 MB XLS).

Dataset S51 YA1S4P2_108 Cy3 = polyA+ RNA nocodazole arrest galactose Mcd1-18Myc in W303; Cy5 = polyA+ reference RNA.
(2.2 MB XLS).

Dataset S52 YA1S4P2_109 Cy3 = polyA+ reference RNA; Cy5 = polyA+ RNA nocodazole arrest glucose Mcd1-18Myc in W303.
(2.2 MB XLS).

Dataset S53 YA1S4P2_110 Cy3 = polyA+ reference RNA; Cy5 = polyA+ RNA nocodazole arrest galactose Mcd1-18Myc in W303.
(2.2 MB XLS).

Dataset S54 YA1S4P2_111 Cy3 = polyA+ RNA nocodazole arrest glucose Mcd1-18Myc in W303; Cy5 = polyA+ reference RNA.
(2.2 MB XLS).

Dataset S55 YA1S4P2_112 Cy3 = polyA+ reference RNA; Cy5 = polyA+ RNA nocodazole arrest glucose Mcd1-18Myc in W303.
(2.2 MB XLS).

Dataset S56 YA1S4P2_114 Cy3 = polyA+ RNA nocodazole arrest galactose Mcd1-18Myc in W303; Cy5 = polyA+ reference RNA.
(2.2 MB XLS).

Dataset S57 YA1S4P2_115 Cy3 = polyA+ RNA nocodazole arrest glucose Mcd1-18Myc in W303; Cy5 = polyA+ reference RNA.
(2.2 MB XLS).

Dataset S58 YA1S4P2_125 Cy3 = polyA+ reference RNA; Cy5 = polyA+ RNA nocodazole arrest glucose Mcd1-18Myc in W303.
(2.2 MB XLS).

Protocol S1 ChIP for Yeast (61 KB DOC).

Protocol S2 Round A/B/C Random Amplification of DNA (37 KB DOC).

### Accession Numbers

The *Saccharomyces* Genome Database (http://www.yeastgenome.org/) accession numbers for the genes and gene products discussed in this paper are *CDC16* (SGDID S0001505), *EBP2* (SGDID S0001655), *GAL2* (SGDID S0004071), Mif2 (SGDID S0001572), *NCE102*/YPR149W (SGDID S0006353), *PCD1* (SGDID S0004141), Pds1p (SGDID S0002520), Rec8 (SGDID S0006211), *ROK1* (SGDID S0003139), *RRS1* (SGDID S0005820), Scc1/Mcd1 (SGDID S0002161), Scc3 (SGDID S0001288), Smc1 (SGDID S0001886), Smc3 (SGDID S0003610), YDL218W (SGDID S0002377), *YDL238C* (SGDID S0002397), *YER179W* (SGDID S0000981), and *YPR006C* (SGDID S0006210).

## Abbreviations

CARC: cohesin-associated region on Chromosome III (C)
CARL: cohesin-associated region on Chromosome XII (L)
ChIP: chromatin immunoprecipitation
ChIP chip: chromatin immunoprecipitation coupled with microarray analysis
DSB: double-strand break
ORF: open reading frame
rDNA: ribosomal DNA
SC: synaptonemal complex
YAC: yeast artificial chromosome
