# Supplementary material for: Genome-Wide Mapping of the Cohesin Complex in the Yeast Saccharomyces cerevisiae
Source: PLoS Biol. 2004 Jul 27;2(9):e259. doi: 10.1371/journal.pbio.0020259 (PMC490026; doi:10.1371/journal.pbio.0020259)
Supplement: Protocol S1 — (61 KB DOC). [file pbio.0020259.sd060.doc]

**CHROMATIN IMMUNOPRECIPITATION (CHIP) PROTOCOL FOR YEAST**

**This protocol is derived from a paper by Miriam Braunstein and is based on work in the Allis lab. The procedure was written by Pamela Meluh and updated by Paul Megee (1/6/03) and Jennifer Gerton (4-6-04).**

References:

Dedon, P. C., J. A. Soults, C. D. Allis, and M. A. Gorovsky. 1991. A simplified formaldehyde fixation and immunoprecipitation technique for studying protein-DNA interactions. *Anal. Biochem.* **197**: 83-90.

Braunstein, M., A. B. Rose, S. G. Holmes, C. D. Allis, and J. R. Broach. 1993. Transcriptional silencing in yeast is associated with reduced nucleosome acetylation. *Genes & Devel.* **7**: 4, 592-604.

**2 Days Before**--Start 5 mL overnight from single colony.

**1 Day Before**--Subculture overnight into 110 mL (large flask for aeration). Dilute so ~2x10^7^cells/mL at convenient time on Day 1, or if a cell cycle arrest will be performed, subculture into 220 mL and have cells at ~5x10^6^cells/mL at the time of addition of alpha factor, HU or NZ.

Reserve Sorvall centrifuge (SS34, 4oC).

Cool large rotor (TMA-3E) for TOMY centrifuge.

**Day 1**

1. When culture is ready, add **formaldehyde** directly to the medium--**1% final concentration**. Calculation: VOL of culture/36 = mL 37% H2C=O. Fix at room temperature with gentle swirling (at least occasionally).

The time of fixation is an experimental variable. For Mif2p, yields of *CEN* DNA in the eventual anti‑Mif2p IP increase with increasing fixation time. For Mif2p, Mcd1-6HA, Mcd1-18MYC, and Smc3-6MYC, 2 h. fixation worked well, although the Smc3-6MYC signal is generally weaker than either Mcd1 signal.

2. Transfer cells to 250 mL conical tubes and spin down in clinical centrifuge. If cells will not be processed until the next day, wash twice with 25 ml cold PBS (transfer to a Nalgene Oak Ridge tube) and resuspend cells in 10 ml PBS and place at 4oC overnight.

3. SPHEROPLAST CELLS. *Volumes are for 100 mL of asynchronous cells.* (I have used the same volumes for 220 ml of staged cells successfully). *Resuspend cells by gently vortexing (avoid pipetting*).

-Resuspend cells in a total of 5 mL **0.1 M TRIS (pH 9.4), 10 mM DTT** (freshly prepared from 1M DTT stock. Place on ice for 15-20 minutes.

-Spin down in Sorvall--5 K, 5 minutes, 4oC. Drain.

-Resuspend cells in 5 mL **HEPES/sorb** to wash. Spin down (5 K, 5 minutes, 4oC). Drain.

-Resuspend in 5 mL **HEPES/sorb** with **0.5 mM PMSF** (add all inhibitors immediately before use). Add 60 µL **1 mg/mL oxalyticase** (can also use 2 mg zymolyase here, as preferred). Incubate at 30oC for 20 minutes with gentle agitation. Check spheroplasting microscopically (cells that are sufficiently spheroplasted will no longer refract light at the edges of the cell). Add additional oxalyticase if needed.

4. When spheroplasting is complete, add 10 mL **PIPES/sorb** with **0.5 mM PMSF**. If spheroplasting of some samples is completed earlier than others, add PIPES/sorb, invert and place on ice until all samples have completed spheroplasting. Spin down immediately--5 K, 5 minutes, 4oC. Drain.

5. WASH SPHEROPLASTS (3x). All manipulations on ice. Use ONLY plastic pipettes to resuspend pellets.

-GENTLY resuspend cell pellet in 5 mL cold **PBS** with **0.5 mM PMSF.** Spin down @ 5 K, 5 minutes, 4oC. Drain.

-Gently resuspend cell pellet in 5 mL cold **Triton X/HEPES** with **0.5 mM PMSF**, **0.8 µg/mL pepstatin A,** and **0.6 µg/mL leupeptin**. Spin down @ 7 K, 7 minutes, 4oC. Drain.

-Gently resuspend cell pellet in 5 mL cold **NaCl/HEPES** with **0.5 mM PMSF**, **0.8 µg/mL pepstatin A**, and **0.6 µg/mL leupeptin.** Spin down @ 7 K, 7 minutes, 4oC. Drain.

-Add 800 µl cold **NaCl/HEPES** with **0.5 mM PMSF**, **0.8 µg/mL pepstatin A**, and **0.6 µg/mL leupeptin** (freshly made) to pellet and gently resuspend using a P1000 with ~1 cm cut from the pipette tip. Transfer resuspended cells to a 2 ml round bottom eppendorf tube. Add another 200 µl of buffer to the Oakridge tube to collect remaining cells and transfer to the 2 ml eppendorf.

-Spin eppendorf tubes at 3000 RPM in a chilled centrifuge and decant.

6. SONICATE SPHEROPLASTS.

-Resuspend cell pellet in 1 mL **SDS lysis buffer** with **1 mM PMSF**, **0.8 µg/mL pepstatin A**, and **0.6 µg/mL leupeptin**. Upon lysis, extract will become quite viscous.

-Sonicate suspension on ice for 10 second intervals, with at least 5 minutes on ice in between, until DNA is in 100-2000 bp range (average size should be â400-500 bp).

For our Branson Sonifier 250: constant output @ 25% power, 6 pulses, 10 sec each.

-Spin in microfuge at maximum speed, 10 minutes, 4oC-10oC (TOMY @ 15K â18,000 x gmax).

7. CHROMATIN SOLUTION

Transfer sup (might seem cloudy from the SDS) to 15 mL Falcon snap-cap tube on ice (expect ~1.1 mL).

Add 10 mL **IP Dilution Buffer** with **1 mM PMSF**, **0.8 µg/mL pepstatin A**, and **0.6 µg/mL leupeptin** (10 mL â9 volumes SDS lysate; final [SDS] â0.1%). Let sit on ice a while (approx. 20 min.).

Spin in TOMY @ 10K (â8,400 x gmax), 10 minutes, 4oC-10oC.

Decant sup into 15 mL conical tube. Place on ice. This is the *chromatin solution*. Set aside 300 *µ*l of chromatin as TOTAL.

8. SET UP IMMUNOPRECIPITATIONS

Aliquot chromatin solution to Eppendorf tube (I use 1.5 mL per IP; ~15-20 O.D.600 equivalents). Add appropriate volume of primary antibody. Incubate overnight 4oC on Nutator. I usually set up duplicate IPs.

Note: Always do **NO Ab** control. Also, if adding competitor antigen, do so several minutes before adding Ab. It may be desirable to denature antigen in SDS first. In this case keep track of the additional SDS and supplement companion IPs to the same final [SDS].

9. BLOCK BEADS WITH BSA. Remove 40 *µ*l of a 50% slurry (50% swelled beads, 50% TE, 0.1% sodium azide) of swelled beads per immunoprecipitation to a 2 ml eppendorf tube. Spin gently in a clinical centrifuge (~2K, 5 minutes), and remove supernatant with a P1000. Wash beads in 1 ml of bead blocking buffer (TE/0.1 % BSA/0.1% sodium azide) and spin as before. Remove supernatant, add another 1 ml of buffer and place on rocking platform with immunoprecipitations.

**Day 2**

10. HARVEST IMMUNE-COMPLEXES

Add ~2 µg (4µl) lambda DNA (*previously sonicated* to 100-2000 bp size range).

Use a razor blade to remove approximately 2 mm from the ends of pippette tips and use these to add 40 µL of **Protein A Sepharose** beads (prepared as 50% slurry in **TE/0.1 % BSA/0.1% azide**) to each tube.

Incubate 1-2 hr. @ room temp. on Nutator.

11. WASH IPs

-Spin down beads (2K, 2 min, 20oC). Remove aliquots of IP sups (æ0.5 mL), if desirable. Aspirate remaining sup.

-Wash beads sequentially with 1 mL of each of the following buffers, nutating beads 3-5 minutes in each buffer:

TSE-150

TSE-150 or TSE-500

LiCl/Det

TE

TE**

**Transfer beads to new Eppendorf with the second TE wash (0.5 mL for transfer + 0.5 mL to rinse old tube and tip).

12. ELUTE IMMUNE-COMPLEXES

-After final IP wash, aspirate as much liquid as possible.

-Add 250 µL **1%SDS/0.1 M NaHCO3**. Vortex briefly, then incubate 15 minutes @ RT on Nutator. Spin down beads. Carefully transfer sup to new Eppendorf, avoiding beads. Be patient here and wait for liquid clinging to pipet tip wall to drain.

-Add another 250 µL **1%SDS/0.1 M NaHCO3** to beads, repeat incubation, and combine sup with first elution. Use a fine pipet tip (gel loading tip) to aspirate liquid remaining in the beads and add to combined sups.

13. REVERSE FORMALDEHYDE CROSSLINKS

Add **5 M NaCl** to samples: 20 µL (1/25 vol.) for eluted immune-complexes (i.e. Pellets)

2.5 µL for 0.3 mL aliquots of Total chromatin solution.

Vortex and briefly spin.

Incubate at 65oC for 8 hr or overnight (12 hours).

**Day 3**

15. Add ~1.5 µL Proteinase K solution (Boehringer Mannheim, 18.6 mg/mL) and 1 ul RNAse (10mg/ml stock). Incubate at 42oC for 1-2 hr.

16. ORGANIC EXTRACTIONS

-Make fresh **phenol:chloroform:isoamyl alcohol** (25:24:1; PCI).

-Extract Pellets *once* with 300 µL **PCI**, then once with 300 µL **CHCl3**. Use PhaseLock gel to generate a physical barrier between aqueous and organic phases. Add PhaseLock to the lids of the tubes before vortexing and spin in an unrefrigerated microfuge (the heat generated during the spinning appears to help PhaseLock form the barrier).

-Extract Totals and Sups *twice* with 400 µL **PCI** and *twice* with **CHCl3**.

17. EtOH PRECIPITATION

Add **glycogen** (60 µg) to Pellets. Add 1/10 volume **3 M NaOAc** and 2 volumes **absolute EtOH** to all samples. Precipitate overnight at -20oC. Note that this level of glycogen may be deleterious if precipitated DNA will be used to probe microarrays. Alternatively, linear acrylamide (10-20 µg) can be used as carrier.

**Day 4**

18. Spin down EtOH precipitates. Wash with **70% EtOH**. Dry briefly in speed vac.

-Resuspend Pellets in 150 µL TE (equivalent to 10 µL chromatin solution per 1 µL).

-Resuspend Totals in TE volume equivalent to 1 µL chromatin solution per 1 µL (e.g. 300 µL TE for Total corresponding to 300 µL chromatin solution).

19. Analyze samples for the presence of various DNA sequences by Slot Blot, Southern, or PCR. Dilute Totals 1:9 in TE before PCR analysis.

*Slot Blot*

Dilute aliquot of each sample in **6xSSC**. Denature at 100oC ~10 minutes. Place immediately on ice. Apply samples to Nytran. Wash 2x with **6xSSC**. UV crosslink filter prior to hybridization.

*Southern Blot*

Usually need to digest at least 1/2 of Pellet samples to see a signal; therefore, aliquot 75 µL to new Eppendorf tube and reduce volume in speed vac. Alternatively, if Southern blot analysis is anticipated, resuspend Pellets in a smaller volume initially. For Totals and Sups, digest an amount of chromatin solution equivalent to 1/5 to 1/10 amount used for Pellets.

For *CEN3*, *Alu*I is the diagnostic digest. Digest for several hours to overnight. Add **RNase** to bluejuice for Totals and Sups prior to loading onto gel. Run a 2.5% agarose gel. Transfer to GeneScreen. (I leave out the HCl treatment when preparing gels for transfer.). UV crosslink prior to hybridization.

*PCR*

I use 3 µL of each sample to program a 50 µL PCR reaction. (Note: Given the volumes used to resuspend various samples, the chromatin solution equivalent for Total is 1/10 that used for Pellet). Controls include no DNA and plasmid or good genomic DNA as a positive control.

Reaction Conditions: DNA (3 µL) Program: 95^o^C, 3 minutes

(in 50 µL) 1x Taq Bfr.

1.5 mM MgCl2 95oC, 30 seconds

0.2 µM each primer Tm-5oC, 45 seconds

0.2 mM dNTPs 72oC, 60 seconds

0.5 µL Taq

Amplify for 26 cycles, total.

72oC, 5 minutes

4oC

Currently, I have obtained the best results by performing 26 cycles of PCR and a dilution of the total of 1:36. Furthermore, I have found that doing a true hot start (adding Taq polymerase to tubes already at 94oC) prevents the production of primer-dimers.

Add 5x Bluejuice. Analyze 1/3 to 1/2 reaction on 2.5% agarose gel or 8% PAGE. Remember to be quantitative!!

NuSieve GTG Agarose Gels

PCR products are run on a 2.5% NuSieve GTG agarose gel (this works well for the typical size range of ChIP PCR products- 150-500 bp. For the large gel trays (20cm x 25cm), 200ml of 2.5% agarose is required. Weigh out agarose and place in a large flask to prevent boiling over during microwaving, and add 1X TBE buffer. The manufacturer recommends using cold buffer, but I have not found this to be critical. Stir the agarose solution for 20 minutes before microwaving. Weigh the flask before microwaving so that water lost during microwaving can be added back to the agarose before pouring. Use the following microwave settings: 3-4 minutes at power level 8, 1-3 minutes at power level 10. Stir the agarose to cool before pouring. Reweigh the flask and add water lost during heating. Just before pouring (I generally pour the gels in the cold), add ethidium bromide to a final concentration of 0.15 µg/ml (3µl of 10 mg/ml stock).

After 30-45 minutes in the cold, place gel in the gel box and add 2 L of 1X TBE containing 0.15 µg/ml ethidium bromide. Let the gel sit for a while before removing combs. I typically run my gels at 140V (approximately 80 mAmps) for 90 minutes. Avoid running gels at voltages that generate currents greater than 90 mA to prevent diffuse bands and heating of the agarose.

**Reagents Needed for ChIP**

37% formaldehyde stock solution

1 M DTT stock

1 M TRIS, pH 9.4 stock

1 mg/mL Oxalyticase stock or 10 mg/mL Zymolyase stock

PMSF, pepstatin A (1 mg/ml in 100% MeOH), leupeptin (1mg/ml in sterile water) stocks

HEPES/sorb *250 mL*:

20 mM HEPES, pH 7.4 5 mL 1 M HEPES

1.2 M sorbitol 150 mL 2 M sorbitol

PIPES/sorb *250 mL*:

20 mM PIPES, pH 6.8 5 mL 1 M PIPES

1 mM MgCl2 0.25 mL 1 M MgCl2

1.2 M sorbitol 150 mL 2 M sorbitol

PBS

Triton/HEPES Wash *250 mL*:

0.25% Triton X-100 3.13 mL 20% Triton X-100

10 mM EDTA 5 mL 0.5 M EDTA

0.5 mM EGTA 0.5 mL 0.25 M EGTA

10 mM HEPES, pH 6.5 2.5 mL 1 M HEPES

NaCl/HEPES Wash *250 mL*:

200 mM NaCl 10 mL 5 M NaCl

1 mM EDTA 0.5 mL 0.5 M EDTA

0.5 mM EGTA 0.5 mL 0.25 M EGTA

10 mM HEPES, pH 6.5 2.5 mL 1 M HEPES

SDS Lysis Buffer *100 mL*:

1% SDS 10 mL 10% SDS (UltraPure)

10 mM EDTA 2 mL 0.5 M EDTA

50 mM TRIS, pH 8.1 5 mL 1 M TRIS

IP Dilution Buffer (for 1:9 dilution) *250 mL*:

0.01% SDS 0.25 mL 10% SDS

1.1% Triton X 100 13.8 mL 20% Triton X 100

1.2 mM EDTA 0.6 mL 0.5 M EDTA

16.7 mM TRIS, pH 8.1 4.2 mL 1 M TRIS, 8.1

167 mM NaCL 8.35 mL 5 M NaCl

Sonicated lambda DNA. Store at 4oC with 0.1% azide.

Protein A Sepharose Bead Buffer *10 mL*:

10 mL TE

10 mg BSA (Fraction V, powder)

100 µl 10 % azide

TSE-150 Wash (â IP conditions) *250 mL*:

0.1% SDS 2.5 mL 10% SDS

1% Triton X-100 12.5 mL 20% Triton

2 mM EDTA 1.0 mL 0.5 M EDTA

20 mM TRIS-HCl, pH 8.1 5.0 mL 1 M TRIS, 8.1

150 mM NaCl 7.5 mL 5 M NaCl

TSE-500 Wash (Optional) *250 mL*:

0.1% SDS 2.5 mL 10% SDS

1% Triton X-100 12.5 mL 20% Triton

2 mM EDTA 1.0 mL 0.5 M EDTA

20 mM TRIS-HCl, pH 8.1 5.0 mL 1 M TRIS, 8.1

500 mM NaCl 25 mL 5 M NaCl

LiCl/Detergent Wash *250 mL*:

0.25 M LiCl 2.65 g LiCl (42.39)

1% NP-40 25 mL 10% NP-40

1% DOC 50 mL 5% DOC

1 mM EDTA 0.5 mL 0.5 M EDTA

10 mM TRIS-HCl, pH 8.1 2.5 mL 1 M TRIS, 8.1

TE, pH 8.0

1% SDS/0.1 M NaHCO3 *50 mL*:

5 mL 10% SDS

5 mL 1M NaHCO3

5x Proteinase K Buffer *10 mL*:

50 mM TRIS, pH 8 0.5 mL 1 M TRIS

25 mM EDTA 0.5 mL 0.5 M EDTA

1.25 % SDS 1.25 mL 10 % SDS

Proteinase K Solution (Boehringer Mannheim, 1413 783), ~18.6 mg/mL

Glycogen stock (20 µg/µl)
