# Supplementary material for: Genome-Wide Mapping of the Cohesin Complex in the Yeast Saccharomyces cerevisiae
Source: PLoS Biol. 2004 Jul 27;2(9):e259. doi: 10.1371/journal.pbio.0020259 (PMC490026; doi:10.1371/journal.pbio.0020259)
Supplement: Protocol S2 — (37 KB DOC). [file pbio.0020259.sd061.doc]

Round A/B/C Random Amplification of DNA Protocol

DeRisi Lab, UC San Francisco, June 2001

<http://derisilab.ucsf.edu/pdfs/Round_A_B_C.pdf>

**This protocol was adapted from Bohlander et al. *Genomics* 13 (1992).**

The goal of this procedure is to randomly amplify any given sample of DNA which

as much representation as possible. It is not a “linear” method, but is useful to

compare relative enrichment between two samples. This protocol has been used

successfully to amplify genomic representations of less than 1ng of DNA.

The protocol consists of three sets of enzymatic reactions. In Round A, Sequenase

is used to extend randomly annealed primers (Primer A) to generate templates for

subsequent PCR. During Round B, the specific primer B is used to amplify the

templates previously generated. Finally, Round C consists of additional PCR

cycles to incorporate either amino allyl dUTP or Cy-dye-coupled nucleotide.

**Caution:** Wear gloves and be careful of contamination as ANY DNA can be

amplified by this protocol. Use filter tips!!! *Always* run a control sample with

water only (no DNA template) to make sure the reagents are not contaminated

with DNA.

Materials

Round A

Sequenase(13 units/µl) US Biochemical cat# 70775

5X Sequenase Buffer

Sequenase Dilution Buffer

3 mM dNTP mix

500ug/ml BSA

0.1 M DTT

40 pmol/µl Primer A: GTT TCC CAG TCA CGA TCN NNN NNN NN

Round B

10X PCR Buffer (500 mM KCl, 100 mM Tris pH 8.3)

25 mM MgCl2

100X dNTPs (20 mM each nucleotide)

5 unit/µl Taq polymerase

100 pmol/µl Primer B: GTT TCC CAG TCA CGA TC

Round C

Same as Round B except use modified dNTP mix

100X modified dNTP mix

25 mM dATP

25 mM dCTP

25 mM dGTP

10 mM dTTP

15 mM aminoallyl-dUTP or Cy-dUTP

(The ratio of aa-dUTP to dTTP can be altered/optimized)

Protocol

1. Round A Reactions

Denature template DNA/primer annealing

As little as 10 nanograms of DNA can be effectively amplified by this protocol.

7 µL DNA

2 µL 5X Sequenase Buffer

1 µL Primer A (40 pmol/µl)

Total Volume = 10 µL

Heat 2 min at 94 °C

Rapid cool to 10 °C and hold 5 min at 10 °C

Add Reaction Mixture to sample:

1 µL 5X Sequenase Buffer

1.5 µL 3 mM dNTP

0.75 µL 0.1 M DTT

1.5 µL 500 ug/µl BSA

0.3 µL Sequenase (13U/µl)

Total Volume = 5.05 µl

Ramp from 10 °C to 37 °C over 8 min.

Hold at 37 °C for 8 min; rapid ramp to 94 °C and hold for 2 min.

Rapid ramp to 10°C and hold for 5 min at 10 °C while adding 1.2 µl of diluted

Sequenase (1:4 dilution)

Ramp from 10 °C to 37 °C over 8 min.

Hold at 37°C for 8 min

Dilute samples with water to final Volume = 60 µl.

2. Round B PCR

Round A Template 15

MgCl2 8

10X PCR Buffer 10

100 X dNTP 1

Primer B(100pmol/µl) 1

Taq 1

Water 63

Round B Cycles:

30 sec 94 °C

30 sec 40 °C

30 sec 50 °C

2 min 72 °C

Run 15-35 cycles, depending on the amount of starting material.

Run 5 µL on 1% agarose gel. A “shmear” of DNA should be present between

500bp –1kb.

It may be necessary to remove aliquots every 2 cycles to check the amplification in

order to optimize the number of cycles. It is best to use the minimal number of

cycles that generates a visible shmear. Make sure there is no DNA in the negative

control lane!

3. Round C

Use 10-15 µL of Round B to seed the Round C reaction:

Round B Template 10-15

MgCl2 8

10X PCR Buffer 10

100X aa-dNTP/cy-dNTP 1

Primer B(100pmol/µl) 1

Taq 1

Water 63-68

30 sec 94 °C

30 sec 40 °C

30 sec 50°C

2 min 72 °C (even longer extension times may improve yield if directly coupling

Cy dyes)

10-25 cycles can be run

If aa-dNTPs were used in Round C, the sample must be desalted (to remove Tris

buffer which interferes with the coupling) prior to dye coupling. Add 400 µL

water to the sample in a Microcon 30, and spin (about 8 min, 12K). Repeat 1X with

500 µl water.

Proceed to Cy-dye coupling as described in the Reverse Transcription protocol.
